# Male Family Caregivers’ Well-Being: Balancing Work and Care for Parents in Japan

**DOI:** 10.1093/geroni/igaa057.1130

**Published:** 2020-12-16

**Authors:** Hiroko Umegaki-Costantini

**Affiliations:** SciencesPo, Paris, France

## Abstract

Despite substantial discussions on caregivers in policy making and academic research, a rapidly emerging new group of middle-aged male family caregivers who often also work are largely understudied. Inadequate understanding of male family caregivers jeopardises not only themselves but also care recipients, other family members and society: better care for older people depends on caregivers’ well-being. I address this gap by understanding how and why gender affects male family carers’ choices of care practices, and how male caregivers as ‘gendered beings’ sustain their well-being while reconciling work and care for their parents. Through in-depth-qualitative narrative interviews based on social anthropology of everyday life with 23 male working family carers in Tokyo in 2019, I find that men select care practices with priority given to familiar tasks deemed appropriately masculine. To perform ‘appropriate’ care for their parents, such men tend to approach care as they do their ‘work’: they ‘work-nise’ care. Such a gendered approach to care practices has important implications for addressing gender in public interventions. Also, I find that for male family working carers, achieving well-being does not mean balance in work, care and family. Rather, for them a source of well-being is from fulfilling filial duty as sons, which, however, requires making an appropriate effort and devoting themselves to some extent to support their parent(s). Thus, such men need to be embedded in a care and employment system that enables them to achieve such well being that, in turn, significantly impacts their care provision.

